# Twitter as an innovation process with damping effect

**DOI:** 10.1038/s41598-021-00378-4

**Published:** 2021-10-28

**Authors:** Giacomo Aletti, Irene Crimaldi

**Affiliations:** 1 ADAMSS Center, Università degli Studi di Milano, Milan, Italy; 2 IMT School for Advanced Studies, Lucca, Italy

**Keywords:** Information theory and computation, Applied mathematics, Statistics

## Abstract

In the existing literature about innovation processes, the proposed models often satisfy the Heaps’ law, regarding the rate at which novelties appear, and the Zipf’s law, that states a power law behavior for the frequency distribution of the elements. However, there are empirical cases far from showing a pure power law behavior and such a deviation is mostly present for elements with high frequencies. We explain this phenomenon by means of a suitable “damping” effect in the probability of a repetition of an old element. We introduce an extremely general model, whose key element is the update function, that can be suitably chosen in order to reproduce the behaviour exhibited by the empirical data. In particular, we explicit the update function for some Twitter data sets and show great performances with respect to Heaps’ law and, above all, with respect to the fitting of the frequency-rank plots for low and high frequencies. Moreover, we also give other examples of update functions, that are able to reproduce the behaviors empirically observed in other contexts.

## Introduction

In our lives we continuously perform actions and these actions can be the repetition of something we have already done in the past or they can be a new experience: we can employ a technology that we already know or we can decide to try a new one, we can listen again a song that we already listened to in the past or we can decide to listen a new song, we can see old friends or we can decide to meet new people and so on. As a consequence, with our actions, we can contribute to diffuse an existing word or idea or product, or we can create a new trend. In particular, thinking about social platform, like *Twitter*, users can diffuse an existing post by means of a “retweet” or a “quote” of it, or they can write a new one.

Understanding the *innovation* process, that is the underlying mechanisms through which novelties emerge, diffuse and trigger further novelties is undoubtedly of fundamental importance in many areas (biology, linguistics, social science and others^1–12^). Novelties can be viewed as first time occurrences of some event and the mathematical object used to model an innovation process is an *urn model with infinitely many colors*, also known as *species sampling sequence*^13–15^. Let $X_1$ be the first observed color, then, given the colors $X_1,\dots , X_t$ of the first *t* extractions, the color of the $(t+1)$-th extracted ball is a new one with a probability $b_t$ which is a function of $X_1,\dots ,X_t$ (sometimes called “birth probability”) and it is equal to the already observed color *c* with probability $p_{c,t}=\sum _{n=1}^t q_{n,t}I_{\{X_n=c\}}$, where $q_{n,t}$ is a function of $X_1,\dots ,X_t$. The quantities $b_t$ and $q_{n,t}$ specify the model: precisely, $b_t$ describes the probability of having a new color (that is a novelty) at time-step $t+1$ and $q_{n,t}$ is the weight at time-step *t* associated to the event *n*, with $1\le n\le t$, so that the probability of having at time-step $t+1$ the “old” color *c* is proportional to the total weight at time-step *t* associated to that color (a reinforcement mechanism, called “weighted preferential attachment” principle). Note that the number of possible colors is not fixed a priori, but new colors continuously enter the system. We can see the urn with infinitely many colors as the space of possibilities, while the sequence of extracted balls with their colors represents the history which has been actually realized.

Although there are only a few explicit prediction rules which give rise to exchangeable sequences, this kind of prediction rules are widely used, because *exchangeability* is a natural assumption in many statistical problems, in particular from the Bayesian viewpoint, and many theoretical results are known for exchangeable sequences^15–17^. We recall that a sequence is said exchangeable if its joint distribution is invariant with respect to permutations that act on only finitely many indices, with the rest fixed. Exchangeability is a powerful assumption, but, in some situations, it could be too restrictive and unrealistic, because it does not take into account the possible causality in the data. Therefore the introduction and study of species sampling sequences, which are not exchangeable, but which still have interesting theoretical properties, is welcome.

The *Blackwell-MacQueen urn* scheme^14,18^ provides the most famous example of exchangeable prediction rule. According to this prediction rule, a new color is observed with probability $b_t=\theta /(\theta +t)$, where $\theta >0$, and an old color is observed with a probability proportional to the number $K_{c,t}$ of times that color was extracted in the previous extractions: $q_{t,n}=1/(\theta +t)$, i.e. $p_{c,t}=K_{c,t}/(\theta +t)$. This is the “simple” preferential attachment rule, also called “popularity” principle. This urn model is also known as Dirichlet process^19^ or as Hoppe’s model^20^ and, in terms of random partitions, it corresponds to the so-called *Chinese restaurant process*^17^. Afterwards, it has been extended introducing an additional parameter and it has been called *Poisson-Dirichlet model*^17,21–23^. More precisely, for the Poisson-Dirichlet model, we have

$$\begin{aligned} &b_t=\frac{\theta +\gamma D_t}{\theta + t}, \qquad q_{n,t}=\frac{1-\gamma /K_{X_n,t}}{\theta + t}, \\&\text{ and } \text{ so } \quad p_{c,t}= \frac{K_{c,t}-\gamma }{\theta + t},  \end{aligned}$$

where $0\le \gamma <1$, $\theta >-\gamma$ and $D_t$ denotes the number of distinct extracted colors until time-step *t*. This model again generates an exchangeable sequence. In^24^, the authors introduce and study a generalization of the Poisson-Dirichlet urn, introducing some random weights so that an old color is observed with a probability proportional to the total weight associated to that color during the previous extractions. The model so obtained does not give rise to an exchangeable sequence anymore, but the generated sequence is a conditionally identically distributed sequence^25^ and so properties usually required in Bayesian statistics are preserved.

From an applicative point of view, as an innovation process, the Poisson-Dirichlet process has the merit to reproduce in many cases the correct basic statistics, namely the Heaps’^26,27^ and the (generalized) Zipf’s laws^28–30^, which quantify, respectively, the rate at which new elements appear and the frequency distribution of the elements.

The *Heaps’ law* states that the number $D_t$ of distinct observed elements (i.e. colors, according the metaphor of the urn) when the system consists of *t* elements (i.e. after *t* extractions from the urn) follows a power law: $D_t\propto t^\gamma$, $0<\gamma \le 1$. Recently, Tria et al.^31–33^ have introduced and studied a new model, called *urn with triggering*, that includes the Poisson-Dirichlet process as a particular case. This model is based on Kauffman’s principle of the adjacent possible^34^: indeed, the model starts with an urn with a finite number $N_0>0$ of balls with distinct colors and, whenever a color is extracted for the first time, a set of balls with new colors is added to the urn. This represents Kauffman’s idea that, when a novelty occurs, it triggers further novelties. Therefore, in the urn with triggering, the space of possible colors expands and it can be seen as an urn with infinitely many colors, where

$$\begin{aligned} b_t=\frac{N_0+\nu D_t}{N_0+\rho t+ a D_t}, \qquad \text{ and }\qquad p_{c,t}=\frac{\rho K_{c,t}+ a-\nu }{N_0+\rho t+ a D_t}, \end{aligned}$$

where $\nu \ge 0$, $\rho > 0$ and $a={{\widetilde{\rho }}}+\nu -\rho +1$ with ${{\widetilde{\rho }}}> -1$. The Poisson-Dirichlet model corresponds to the case $a=0$ (taking $\theta =N_0/\rho$ and $\gamma =\nu /\rho \in [0,1)$). In general, the sequences generated by the urn with triggering are not exchangeable. Moreover, while the Poisson-Dirichlet process can predict only a sub-linear power law behavior for the number of distinct observed colors/elements (i.e. Heaps’ law with $\gamma <1$), the urn with triggering is able to provide also a linear growth for it (i.e. Heaps’ law with $\gamma =1$): precisely, we have $D_t\propto t^{\nu /\rho }$ for $0<\nu <\rho$, $D_t\propto t/\ln (t)$ for $\nu =\rho$, $D_t\propto t$ for $\nu >\rho$ and, finally, $D_t\propto \ln (t)$ for $\nu =0$. A different model able to reproduce all the Heaps’ exponents in [0, 1] is the one proposed in^35^, where the innovation process is described as an edge-reinforced random walk on an underlying network of relations among concepts (nodes). The topology naturally plays a key role in this last model so that it results useful only in the cases where the network can be well reconstructed from data (e.g. innovations in a scientific discipline by means of the analysis of scientific publications).

The *Zipf’s law* states an inverse proportionality between the frequency and rank of the considered quantities. Let us consider a generic sequence of elements (colors) and count the number of occurrences of each element. Now, suppose one repeats the same operation for all the distinct elements in the sequence, and ranks all the elements according to their frequency of occurrence (rank $r=1$ corresponds to the most frequent color, the rank $r=2$ correspond to the second most frequent color and so on, the higher the rank, the less frequent the color) and plots them in a graph showing the number of occurrences versus the rank. The (generalized) Zipf’s law affirms a frequency-rank distribution of the form $z(r)\propto r^{-\alpha }$, with $0<\alpha <+\infty$ (the strict Zipf’s law refers to $\alpha =1$). This property is used in theoretical analyses^36^ or it is seeked by mean of various mechanisms^37–42^. The urn with triggering asymptotically (i.e. for large times) satisfies the Zipf’s law when $\nu \ne 0$, with the relationship between the Heaps’ and Zipf’s exponents given by $\gamma = 1/\alpha$ when $\gamma =\nu /\rho <1$.

However, in some cases the frequency-rank plots observed in empirical applications are far from showing a pure power law behavior and the above relation between the Heaps’ and Zipf’s exponents holds asymptotically and only when looking at the tail of the frequency-rank plot, i.e., for large ranks, that correspond to small frequencies (rare elements)^32,33,43–48^. To the best of our knowledge, few works have tried to explain the empirical frequency-rank plots in the part of small ranks, i.e. for high frequencies, where they typically deviate from a power law behavior. The papers^43,46,48^ provides examples where the frequency-rank plot exhibits a power law behavior with two different scaling exponents: $z(r)\propto r^{-\alpha _1}$ for $r< \xi$ and $z(r)\propto r^{-\alpha _2}$ for $r>\xi$, where $\alpha _1$ is typically equal to 1. In^46^ this empirical finding is achieved by means of a generative stochastic model based on the existence of two different classes of words: a finite number of core words, which are more frequently used and do not affect the probability of a new word to appear and the remaining infinite number of non-core words, which are significantly less used and, once used, reduce the probability of a new word to be employed in the future. In^48^ no generative models are provided, but the two scaling regimes of the word frequency distributions are again explained using two categories of words: the specialized words in the “unlimited lexicon” are not universally shared and are employed less frequently than the words in the “kernel lexicon”. In^44^, the authors introduce a variant of the Simon’s model^41^ (which is a species sampling sequence where $b_t$ is equal to a constant and the probability of observing an old color is proportional to the number of times that color was extracted in the previous extractions) that also incorporates a long-term memory or aging component: precisely, at a time step *t*, a new element appears with probability *b*, whereas with probability $(1-b)$, an old element is chosen, going back in time by *n* time steps with a probability $Q_t(n)$ that decays as a power law. This generative stochastic model is able to fit with high accuracy the observed frequency-rank plots in the collaborative tagging context. Finally, in^47^, the authors observe an exponential decay in the frequency-rank plot and this fact is ascribed to the limited dictionary size (indeed, they consider Chinese, Japanese and Korean languages). In order to reproduce this empirical finding, they provide a generative model based on a finite dictionary size of distinct characters.

In this work we are going to show that the deviations from the Zipf’s law in the empirical frequency-rank plots (in particular, in the part of small ranks) can be explained adding a *“damping” effect* in the urn with triggering. More precisely, we generalize the urn with triggering model by the introduction of a function *F* that drives the update mechanism of the number of balls of the same color of the extracted one when it is of an “old” color. In the standard model, this function is linear so that it generates a power law behavior of the frequency-rank plot (Zipf’s law), that usually matches the empirical ones only in the part of rare elements (i.e. large ranks). Instead, if we take the function *F* linear until a certain point and then still linear but with a smaller slope or sub-linear (for instance, the square root), then we obtain a frequency-rank plot closer to the empirical ones also in the part of high frequencies. This fact can be seen as a damping effect on the old elements: the number of balls of an old color increases linearly with the number of times it is extracted until a certain threshold, then it increases slower. Our unique general model is able to reproduce the empirically observed power law behavior with two different scaling exponents mentioned above and also other kinds of curves, observed in real data sets. Indeed, given the function *g* that fits the empirical frequency-rank plot (see eq. (9)), we are able to find the corresponding function *F* of the proposed generative model (see eq.(3)). This is a very useful result for applicative purposes, since in applications one usually observes and tries to fit the empirical frequency-rank plot. Further, we have shown how to obtain the asymptotic behavior of the number $D_t$ of distinct observed elements starting from the function *g* and we have employed this methodology with some specific functions *g*, coming from empirical studies. The obtained theoretical results are supported by simulations.

We apply the proposed model to some data sets from the social platform *Twitter*. The main mechanism in Twitter leading content diffusion is the possibility for users to “re-tweet”, reply or quote the post sent by others. Therefore, ordered sequences of posts can be seen as generated by an urn model with infinitely many colors: a new color is associated to a new tweet, while the extraction of an old color corresponds to a re-sharing (by means of a re-tweet or a quote or a reply to) of an old post. The update function *F* rules the probability of a generic posted tweet to be re-shared. For all the considered data sets, we observe the same damping effect on the old elements: the update function *F* grows linearly until a certain threshold, then it increases sub-linearly, precisely according to the square root. Moreover, we empirically verify the linear growth of the variable $D_t$, which agrees with the proven theoretical result. We refer to^49^ for a survey on information diffusion in on-line social networks. In^50^ the authors face the problem to predict the future time evolution of the popularity of a certain tweet and, in particular, to estimate the final number of retweets of that given tweet (see also the reference therein for other works related to the same question). In^51,52^ the focus is instead on the “retweet graph”, which is the graph of users who participated in the discussion of a specific topic and where a directed edge indicates that a user retweeted a tweet of another user.

Finally, we explain our choice of the term “damping” with respect to the other terms “saturation” and “aging” employed in literature. The term “saturation” is typically used when the probability of observing a new element goes to zero in such a way that $D_t$ converges to a certain finite value^47^; while, the term “aging” refers to time in the sense that the probability of having the repetition of a certain old element decreases with the difference between the present time and the last time of observation of that old element^44^. The effect that we model refers to a damping factor in the probability of a repetition of an old element: using the metaphor of the urn, the number of balls of a given color in the urn increases with the number of times that color has been extracted according to a suitable update function that exhibits two different speeds, one before a certain threshold and a lower one after the threshold. Therefore, it obviously differs from the above saturation effect and it is related in some sense to the age of the elements, but not directly and so it is not exactly the same of the above recalled aging effect.

Summing up, the contribution of the present paper is twofold. First, we introduce a general and flexible model for innovation processes that is able to generate frequency-rank plots that are not pure power law. Hence, this work could result interesting for researchers who work in various contexts of innovation theory. Second, we analyze some Twitter data sets, that shows the presence of a damping effect, perfectly explained by means of the proposed model with a suitable update function. Therefore, this work could be of interest for who are involved in the study of Twitter activity.

## Results

We firstly introduce the model, using the metaphor of the urn, and we state the main related results. Then we show the empirical results.

**Model.** Given an increasing function *F* defined on ${\mathbb {\mathbb {N}}}\setminus \{0\}$ with $F(1)>0$, the model works as follows. An urn initially contains balls of $N_0>0$ different colors. For each color, we have one ball. Then, at each time step *t*, a ball is drawn at random from the urn, its color is registered in a sequence ${\mathcal {S}}$ and:

- if the color of the extracted ball is a new one, that is it appears for the first time in ${\mathcal {S}}$ (it corresponds to the realization of a novelty), then the number of balls of the extracted color in the urn becomes $F(1)>0$ and we add $\nu +1$ (with $\nu \ge 0$) distinct balls of different new colors, that is of colors not yet present in the urn (precisely, we add one ball for each new color);
- if the color *c* of the extracted ball is already present in ${\mathcal {S}}$ and $K_{c,t}$ denotes the number of times the color *c* was extracted until time step *t* (included), the number of balls of color *c* in the urn becomes $F(K_{c,t})>0$.

The fact that the *update function* *F* is increasing means that we have a *reinforcement mechanism*: the larger the number of times the color *c* has been extracted, the larger the number of balls of color *c* in the urn. Moreover, the addition of a set of balls with new colors in the urn whenever a color is extracted for the first time represents Kauffman’s *principle of the adjacent possible*^34^, i.e.  the idea that, when a novelty occurs, it triggers further novelties.

If $X_{t+1}$ is the color of the extracted ball at time step $t+1$, $D_t$ is the number of different colors extracted until time step *t*, $N_t$ is the number of different colors in the urn until time step *t* and $T_t$ is the total number of balls in the urn at time step *t*, we have:

$$ \begin{aligned} N_t&=N_0+(\nu +1)D_t\,\\ T_t&=\sum _{j=1}^{N_t} F(K_{j,t})= N_0+\nu D_t+\sum _{j\in {\mathcal {S}}} F(K_{j,t})  \end{aligned}$$

(the sum $\sum _{j\in {\mathcal {S}}}$ denotes the sum on the $D_t$ different colors in ${\mathcal {S}}$) and

1 $$\begin{aligned} b_t=P(X_{t+1}=\hbox {{new}}\,|\,X_1,\dots ,X_t)= \frac{(N_t-D_t)}{T_t} = {\left\{ \begin{array}{ll} 1\qquad &{}\text{ for } t=0\\ \frac{N_0+\nu D_t}{N_0+\nu D_t+\sum _{j\in {\mathcal {S}}} F(K_{j,t})} \quad &{}\text{ for } t\ge 1\,. \end{array}\right. } \end{aligned}$$

Moreover, if *c* denotes an *old* color, we have for each $t\ge 1$

2 $$\begin{aligned} p_{c,t}=P(X_{t+1}=c\,|\,X_1,\dots ,X_t)= \frac{F(K_{c,t})}{T_t}= \frac{F(K_{c,t})}{N_0+\nu D_t+\sum _{j\in {\mathcal {S}}} F(K_{j,t})}\,. \end{aligned}$$

The quantities $b_t$ and $p_{c,t}$ drive the model. The model parameters $N_0$ and $\nu$ can be any real positive numbers with $\max \{N_0,\nu \}>0$. The update function *F* can be any increasing function defined on the strictly positive integer numbers and with values in $(0,+\infty )$. For instance, the standard urn model with triggering^31–33^ corresponds to the update function *F* defined as $F(x)= {\widehat{\rho }}+\rho (x-1)$, with ${\widehat{\rho }}={\widetilde{\rho }}+1>0$ and $\rho >0$.

For the case $\nu >0$, when we assume a dependence in the frequency-rank plot of the form $g(z(r)) = -a \ln (r) + b$, with an invertible differentiable function *g* and a constant $a>0$, we obtain (see Section 3.2 for the computations) the relation

3 $$\begin{aligned} F(x)\approx \frac{a\nu }{g'(x)}. \end{aligned}$$

This relation is not an exact equality, because it is due to (8) and (10), which have been obtained by approximation. From the applicative point of view, relation (3) is of fundamental utility because it allows us to guess the right update function *F* in the model by means of the function *g* that fits well the empirical frequency-rank plot. For instance, for the standard urn with triggering, we have $g=\ln$ with $a=\rho /\nu$ and, indeed, we have $F(x)={{\widehat{\rho }}}+\rho (x-1)\approx \rho x=a\nu x$ for large *x* and so (3) is satisfied. More generally, whenever we have a Zipf’s law, that is $g=\ln$ in (9), we get that, according to (3), we have to choose a linear update function *F* in the model. Regarding the asymptotic behavior of the number $D_t$ of distinct elements in ${\mathcal {S}}$ as a function of its length *t*, we note that

4 $$\begin{aligned} t=\sum _{r=1}^{D_t} z(r)\approx \int _1^{D_t}z(r)dr \end{aligned}$$

and so the behavior of $D_t$ can be obtained starting from the frequency-rank function $r\mapsto z(r)$. In the Section 3.3, we derive the asymptotic behavior of $D_t$ starting from different kinds of function *g*.

**Figure 1:**
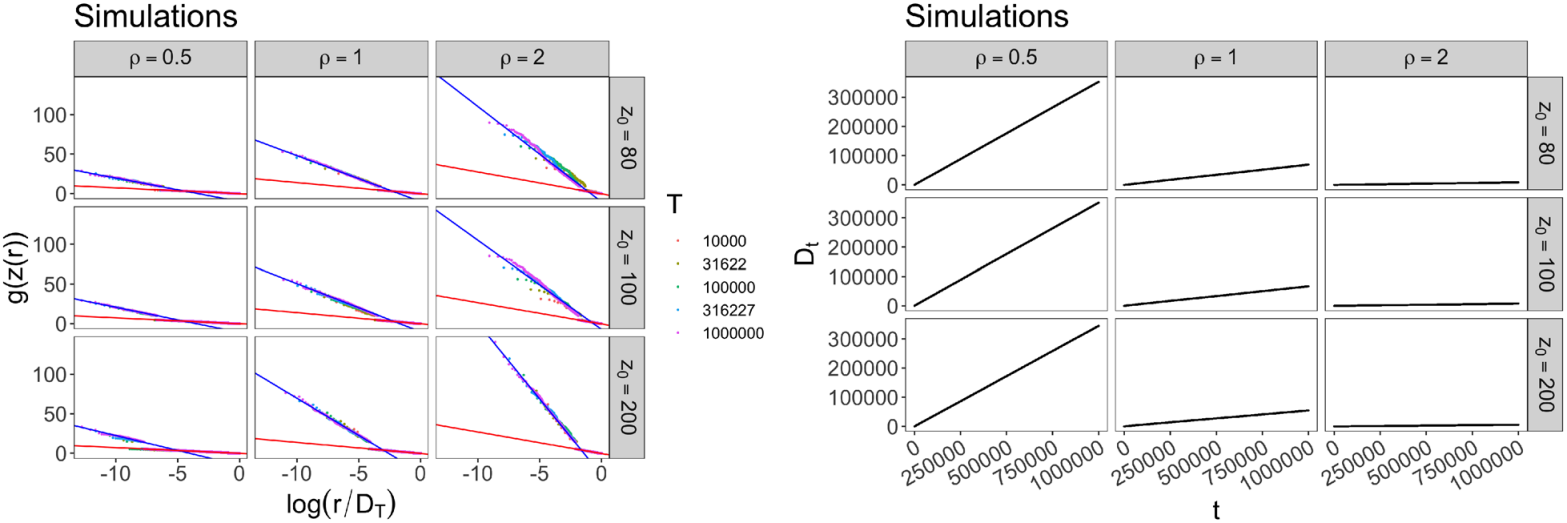
Simulations for case (5) with $N_0=2$, $\nu =0.75$ and different values of $\rho$ and $z_*=z_0$: (Left) Frequency-rank plot in two different scales: log-sqrt before a certain $R_*$ and log-log after $R_*$. The different colors of the dots correspond to different quantities of data taken for depicting the plot. (Right) Behavior of $D_t$.

**Figure 2:**
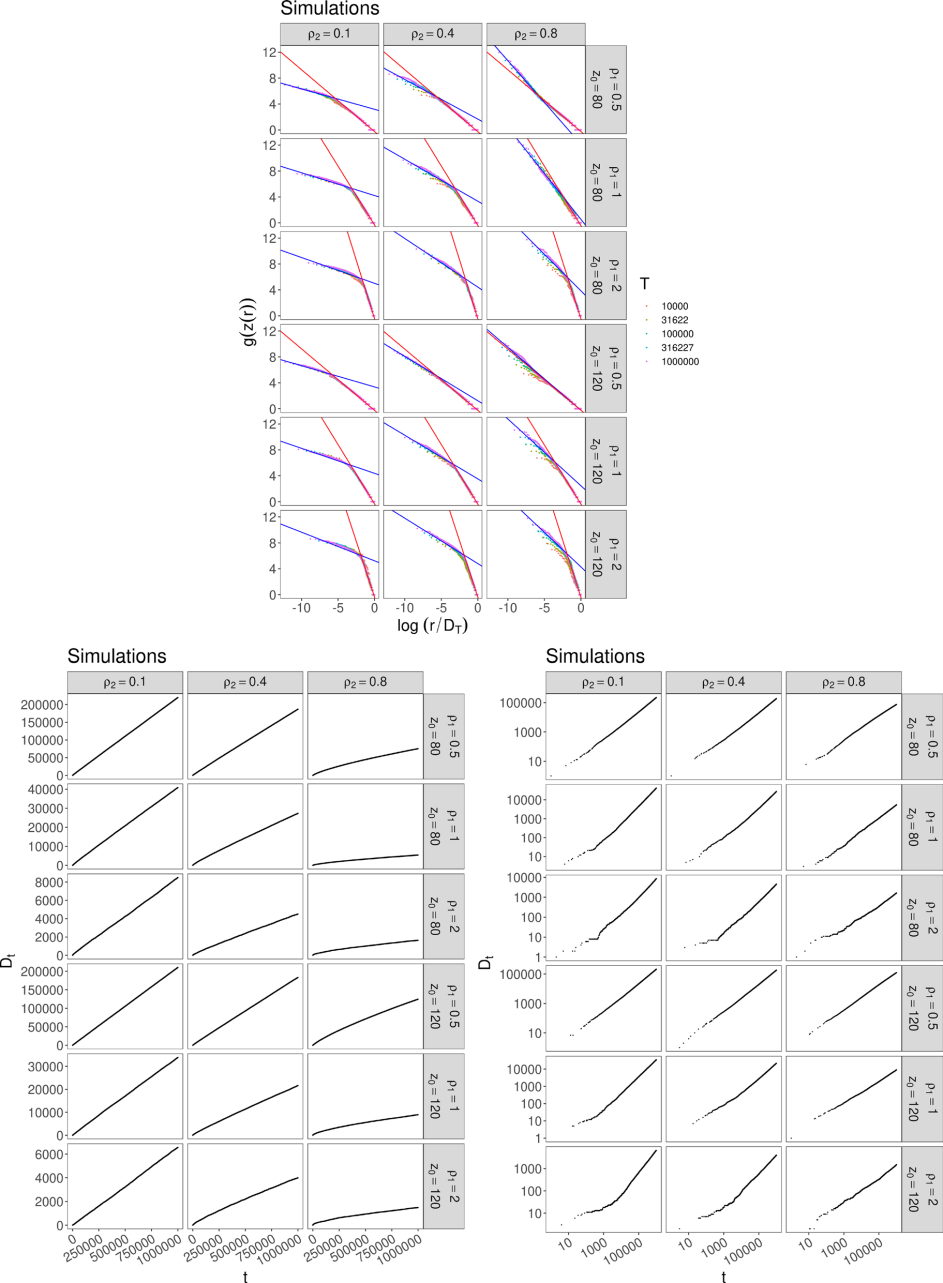
Simulations for case (6) with $N_0=2$, $\nu =0.75$ and different values of $\rho _1,\,\rho _2$ and $z_*=z_0$: (Up) Frequency-rank plot in log-log scale. The different colors of the dots correspond to different quantities of data taken for depicting the plot. (Below) Behavior of $D_t$: the plot on the left shows a linear growth of $D_t$ (Heaps’ law with exponent 1) when $\rho _2/\nu <1$ and the plot on the right shows a power-law growth (Heaps’ law with exponent smaller than 1) when $\rho _2/\nu >1$.

**Figure 3:**
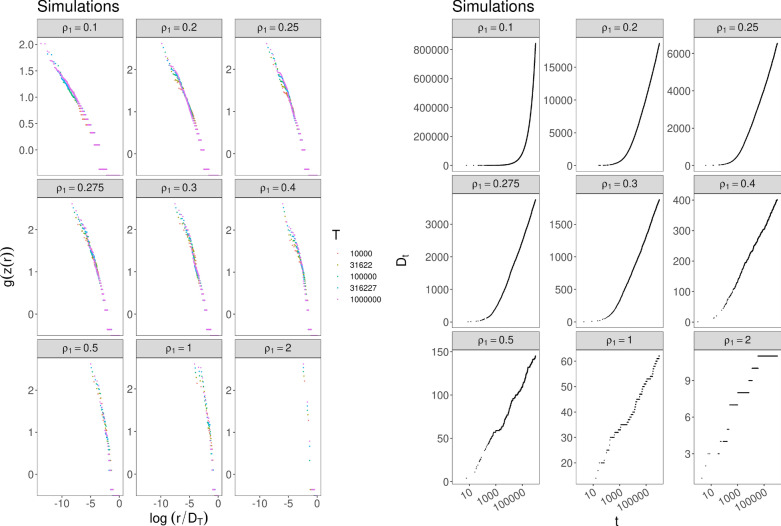
Simulations for case (7) with $N_0=2$, $\nu =0.75$ and different values of $\rho _1$: (Left) Frequency-rank plot with $g(z)=\ln (\ln (z))$. The different colors of the dots correspond to different quantities of data taken for depicting the plot. (Right) Behavior of $D_t$.

In Figs. 1, 2 and 3, we show the simulations of the model with $N_0=2$, $\nu =0.75$ and different kinds of update function *F*. The relation (3) and the other theoretical results described in the Methods Section are supported by these simulations. More precisely, in Fig. 1 we exhibit the results for

5 $$\begin{aligned} F(x)={\left\{ \begin{array}{ll} \rho x \quad &{}\text{ for } 1\le x<z_*\\ \rho \sqrt{z_*x} \quad &{}\text{ for } x\ge z_*\,, \end{array}\right. } \end{aligned}$$

for different values of $\rho$ and $z_*$. The model with this function *F* gives rise to a dependence structure in the frequency-rank plot described by $g(z) = \ln (z)$ before $z_*$ and $g(z)=\sqrt{z}$ after $z_*$, and to a linear growth of $D_t$, that is a Heaps’ law with exponent 1. (See Subsec. 3.3.1 for technical details).

In Fig. 2 we exhibit the results for

6 $$\begin{aligned} F(x)={\left\{ \begin{array}{ll} \rho _1 x \quad &{}\text{ for } 1\le x<z_*\\ \rho _2x+ (\rho _1-\rho _2)z_* \quad &{}\text{ for } x\ge z_*\,, \end{array}\right. } \end{aligned}$$

for different values of $\rho _1,\,\rho _2$ and $z_*$. The model with this function *F* gives rise to a Zipf’s law with two different coefficients and to different kinds of behavior for $D_t$, depending on the value $\rho _2/\nu$. (See Subsec. 3.3.2 for technical details).

Finally, in Fig. 3 we exhibit the results for

7 $$\begin{aligned} F(x)=\rho _1 x\ln (x+1) \end{aligned}$$

for different values of $\rho _1$. The model with this update function *F* gives rise to an exponential decay of the frequency-rank function *z*(*r*) (that corresponds to $g(z)=\ln (\ln (z))$) and to a logarithm growth of $D_t$. (See Subsec. 3.3.3 for technical details). It is worthwhile to note that these behaviors for *z*(*r*) and $D_t$ can be achieved also by the standard urn with triggering with $\nu =0$ (see^32^). However, the two models are completely different: for the standard model, the assumption regards the parameter $\nu$ that rules the probability $b_t$ of a new color, while the number of balls of an old color increases linearly according to a “free” coefficient; whereas, for the proposed new model, the assumption regards the update function *F* that drives the growth of the number of balls of an old color in the urn and the parameter $\nu$ can be whatever.

**Empirical results.** Two data sets have been collected from the Twitter platform, using the official API to stream the exchange of messages on several topics:

- *Italy, Migration debate*
  Data were collected through the Filter API since 23rd of January to 22nd of February 2019 and targeted the Italian debate on migration. The total number of posts is $T=1\,066\,677$. More details on the data set can be found in^53^.
- *Italy, COVID-19 epidemic*
  The data set covers the period from February 21st to April to 20th 2020, including tweets in Italian language. The keywords used for the query are relative to the COVID-19 epidemic. The total number of posts is $T=4\,580\,781$. More details on the data set can be found in^54^.

Using the metaphor of the urn, the extractions correspond to the publication of posts on Twitter. Therefore the sequence ${\mathcal {S}}$ of colors is constructed looking at the posts ordered by their time-stamps. The color of the ball tells if the post is a new tweet (that is a novelty) or a re-tweet/quote/reply (that is a repetition): a new color is associated to a new tweet; while an old color corresponds to a re-sharing (by a re-tweet or a quote or a reply) of an old post. More precisely, in the latter case, we register in ${\mathcal {S}}$ the color of the original message: for instance, given $t_1<t_2<t_3$, if at time $t_3$ we have the quote of the post published at time $t_2$, which is a retweet of the post at time $t_1$, we register at positions $t_2$ and $t_3$ the same color of $t_1$. For the sequence obtained from the data set regarding the migration debate, the number of observed distinct colors is $D_T=210\,190$ and the maximum number of times a given color is repeated is $z_{max}=3\,694$. For the sequence obtained from the data set regarding the COVID-19 epidemic, the number of observed distinct colors is $D_T=1\,447\,623$ and the maximum number of times a given color is repeated is $z_{max}=4\,818$.

**Figure 4:**
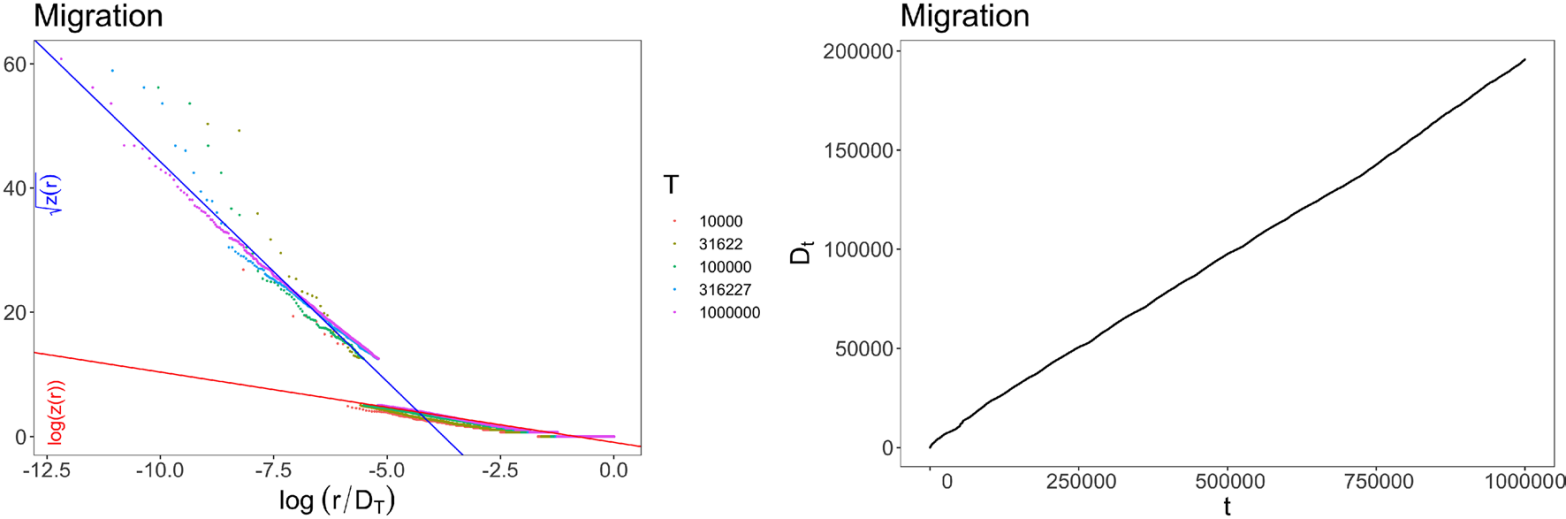
Migration: (Left) Frequency-rank plot in two different scales: log-sqrt before a certain $R_*$ and log-log after $R_*$. Parameters (see Sections 3.3.1 and 3.4 for details): $a_1=7.07$, $a_2=1.13$, $R_*\in [-5.97,-5.19]$, that give $\rho /\nu =a_2=1.13$ and $z_*=4(a_1/a_2)^2= 156.58$. The different colors of the dots correspond to different quantities of data taken for depicting the plot. (Right) Behavior of $D_t$.

**Figure 5:**
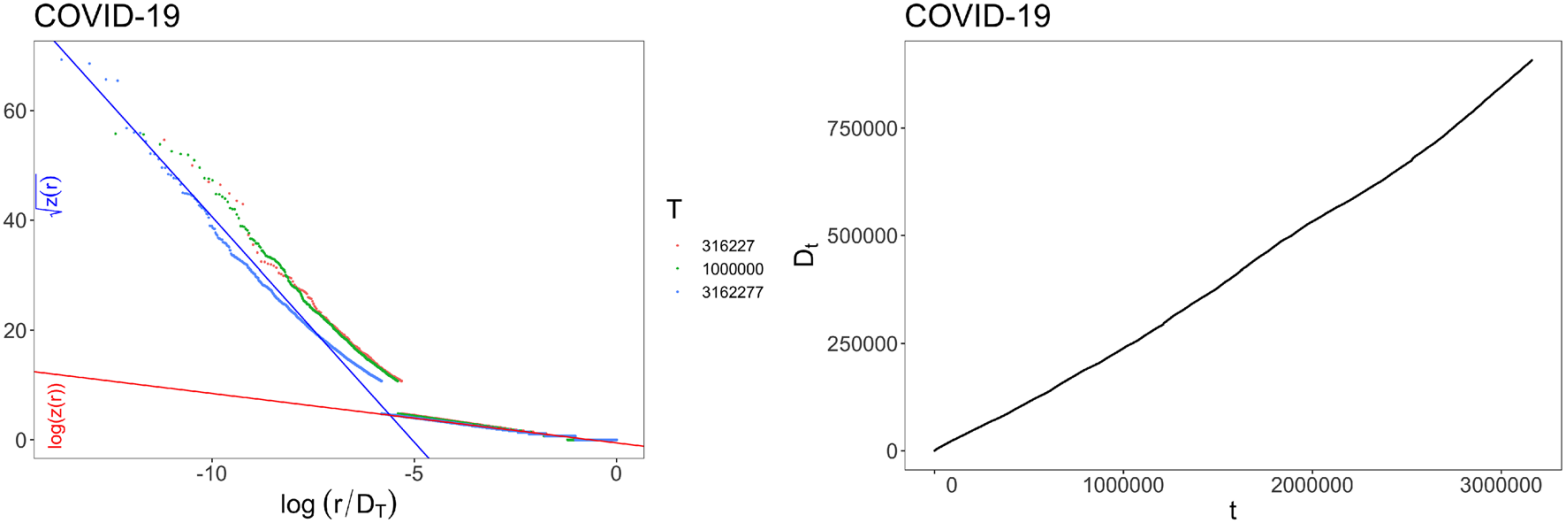
COVID-19: (Left) Frequency-rank plot with two different scales: log-sqrt before a certain $R_*$ and log-log after $R_*$. Parameters (see Sects. 3.3.1 and 3.4 for details): $a_1=8.68$, $a_2=0.895$, $R_*\in [-7.44,-6.80]$, that give $\rho /\nu =a_2=0.895$ and $z_*=4(a_1/a_2)^2= 376.23$. The different colors of the dots correspond to different quantities of data taken for depicting the plot. (Right) Behavior of $D_t$.

**Figure 6:**
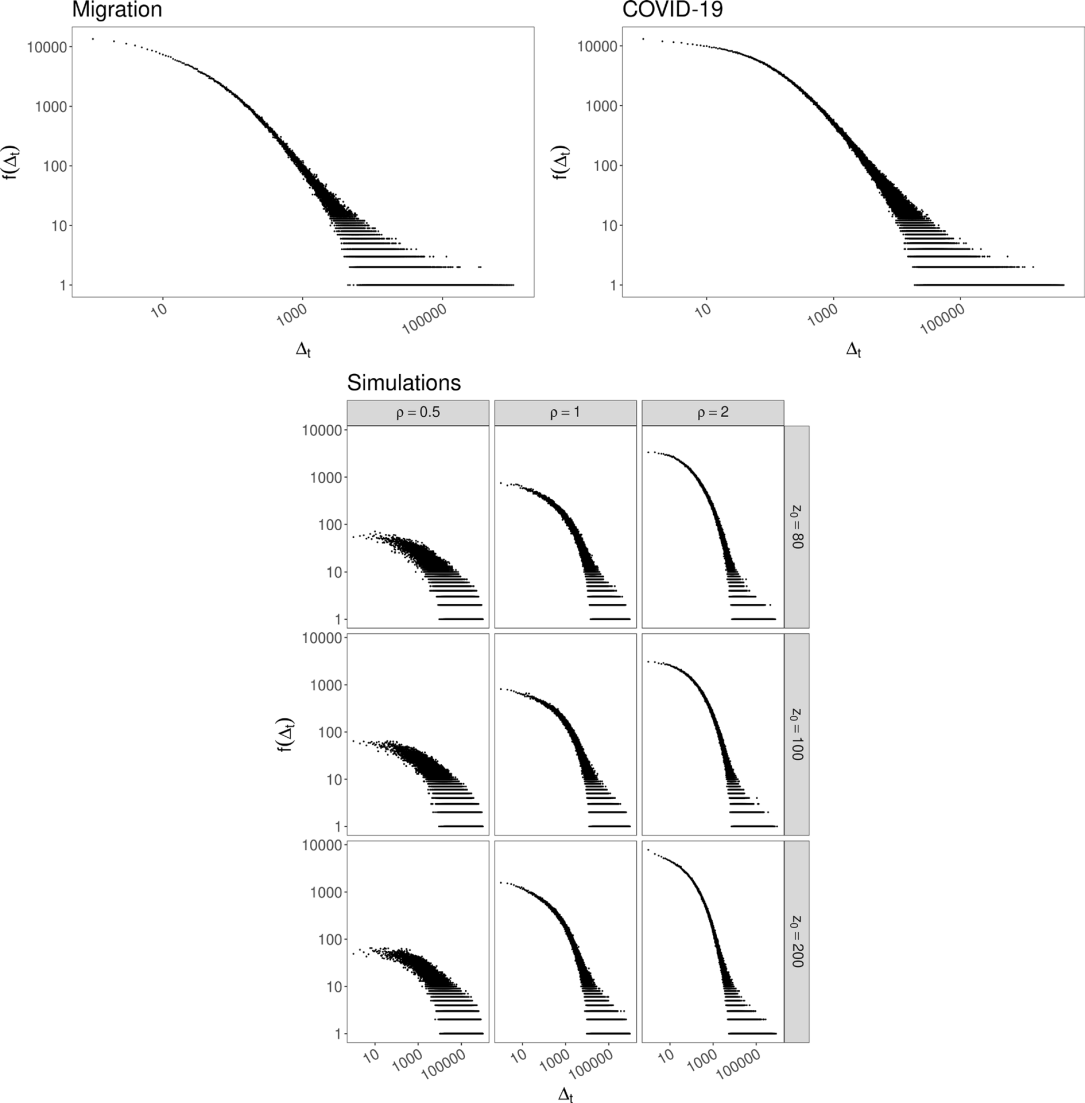
Frequency distribution of inter-event time steps between pairs of consecutive occurrences of the same color: (Up, Left) Migration; (Up, Right) COVID-19; (Below) Simulations of the model with *F* given by (13), $N_0=2$, $\nu =0.75$, ${\widehat{\rho }}=\rho$ and different values of $\rho$ and $z_*=z_0$.

When a new tweet has been sent, the addition of $\nu +1$ balls of new distinct colors can be seen as the potential new tweets that the posted tweet may generate. Hence the parameter $\nu$ is related to the ability of a generic new tweet to give rise to future new tweets. On the other hand, the update function *F* rules the probability of a generic posted tweet to be re-shared (with a retweet or a quote or a reply). For all the considered data sets, we observe the same damping effect on the old elements: the update function *F* increases linearly until a certain threshold, then it increases sub-linearly as the square root. Indeed, looking at the empirical frequency-rank plot, we observe a dependence structure given by $g(z) = \ln (z)$ before a certain threshold $z_*$ and $g(z)=\sqrt{z}$ after $z_*$ (see the left panels in Figs. 4 and 5), that corresponds to the update function *F* in the model described in (13). Moreover, we verify (see the right panels in Figs. 4 and 5) the linear growth of the number $D_t$ of distinct observed tweets, which agrees with the proven theoretical result (see Subsec. 3.3.1). Finally, Fig. 6 shows that for both data sets the frequency distribution $f(\Delta _t)$ of inter-event time steps $\Delta _t$ between pairs of consecutive occurrences of the same color in ${\mathcal {S}}$ exhibits a behavior similar to the one obtained by simulations of the model with *F* given by (13).

## Discussion

The innovation models introduced so far satisfy the Heaps’ law, regarding the rate at which novelties appear, and the Zipf’s law, that states a power law behavior for the frequency distribution of the elements. However, there are empirical cases far from showing a pure power law behavior and such a deviation is present for elements with low ranks, that is with high frequencies. In this work we explain such deviations from the Zipf’s law by adding a suitable *damping effect* in the urn with triggering model. More precisely, we generalize the standard urn with triggering^31–33^ by the introduction of a function *F* that drives the *update mechanism* of the number of balls of the same color of the extracted one when it is of an old color. Indeed, if we take the update function *F* linear until a certain point and then still linear but with a smaller slope or sub-linear, then we obtain a frequency-rank plot closer to the empirical ones also in the part of high frequencies. This means that the number of balls of an old color increases linearly with the number of times it is extracted until a certain threshold, then it increases slower, that is we have a damping factor in the updating of the urn.

Given the function *g* that fits the empirical frequency-rank plot (see eq. (9)), we are able to find the corresponding update function *F* of the proposed generative model (see eq.(3)). This is a very useful result for applicative purposes, since in applications one usually observes and tries to fit the empirical frequency-rank plot. Further, we have shown how to obtain the asymptotic behavior of the number $D_t$ of distinct observed elements starting from the function *g* and we have employed this methodology with some specific functions *g*. The obtained theoretical results are supported by simulations.

We have applied the proposed model to some data sets from the social platform Twitter, where the update function *F* rules the probability of a generic posted tweet to be re-shared. For all the considered data sets, we observed the same damping effect on the old elements: the update function *F* grows linearly until a certain threshold, then it increases sub-linearly, precisely according to the square root. Moreover, we empirically verified the linear growth of the variable $D_t$, which agrees with the proven theoretical result. We would like to point out that the analyzed data sets include a huge number of posts, but they cover a short period of time (one or two months). Therefore, a possible research line for the future could be to investigate if data sets collected over longer periods exhibit the same behavior or are well fitted with a different update function *F*. For instance, we can generalize the proposed model taking into account a time-dependent update function. Moreover, both topics for which data were collected are such that there was considerable public interest on the topics during the period of collection. Hence, it could be interesting to consider topics that emerge suddenly (such as a natural disaster) but interest on the topic does not stay for a long period. To this regard, we think that the model will be again able to reproduce the phenomenon taking an update function that increases with two different rates: again, a damping effect, such as in the data sets analyzed in the present paper, but probably with a more heavily damping in the growth rate of the update function.

Finally, we underline that the proposed model provides a general framework that is able to explain also the power law behavior with two different scaling exponents observed in^43,46,48^ and other kinds of empirically observed curves (e.g.^47^). Therefore, it results a very flexible generative model that could perfectly reproduce the frequency-rank plot for low and high ranks, together with the behavior of $D_t$, in many contexts. It could be interesting to consider in the future other data sets, related to different contexts and possessing different characteristics, and exploit the proposed model with other update functions.

## Methods

Take $\nu >0$ and assume *F* to be extended with continuity on the whole $[1,+\infty )$ and in such a way that it is differentiable everywhere except in a finite number of points.

### Relation between *F* and the frequency distribution

For each $k\ge 1$, we denote by $Q_{k,t}$ the number of colors *c* in ${\mathscr{S}}$ with $K_{c,t}=k$ and we set $p_k=\lim _t Q_{k,t}/D_t$. The family $(p_k)_k$ is the (stationary) frequency distribution.

We have $D'_t=b_t=\frac{N_0+\nu D_t}{T_t}$ and so we can write $T_t\approx \frac{\nu D_t}{D'_t}$. Moreover, we have $\sum _{k\ge 1} Q_{k,t}=D_t$ and we can write the following master equation for $Q_{k,t}$:

$$ \begin{aligned} \frac{\partial Q_{k,t}}{\partial t}&= -\frac{Q_{k,t}F(k)}{T_t} +\frac{Q_{k-1,t}F(k-1)}{T_t}= -\frac{(Q_{k,t}-Q_{k-1,t})F(k)+Q_{k-1,t}(F(k)-F(k-1))}{T_t} \\&\approx - \frac{1}{T_t}\frac{\partial F(k)Q_{k,t}}{\partial k} \approx -\frac{D'_t}{\nu D_t}\frac{\partial F(k)Q_{k,t}}{\partial k}\,.  \end{aligned}$$

Using the asymptotic relations $Q_{k,t}\approx p_k D_t$ and $\frac{\partial Q_{k,t}}{\partial t}\approx D'_t p_k$, from the above relation we get

$$\begin{aligned} p_k\approx -\frac{1}{\nu }\frac{d[F(k)p_k]}{dk}\,. \end{aligned}$$

Since $\frac{d[F(k)p_k]}{dk}=F'(k)p_k+F(k)p'_k$, we obtain

$$\begin{aligned} \frac{p'_k}{p_k}\approx -\frac{\nu }{F(k)}-\frac{F'(k)}{F(k)}\,. \end{aligned}$$

When 1/*F* has primitive function *H*, this equation has solution $\ln (p_k)\approx -\ln (F(k))-\nu H(k) + C$, that is

8 $$\begin{aligned} \ln (p_k) \approx - \ln (F(k)) - \nu \int _{k_0}^{k} \frac{1}{F(x)}dx +C'\,, \end{aligned}$$

where *C* and $C'$ denote suitable constants. The above relation provides the relationship between the function *F* in the model and the frequency distribution. For instance, for the standard urn with triggering, we get $\ln (p_k)\approx -(1+\nu /\rho )\ln \big ({{\widehat{\rho }}}+\rho (k-1)\big )+C''$, that is $p_k\propto k^{-(1+\nu /\rho )}$ for large *k* (see also^32^).

### Relation between *F* and the frequency-rank plot

Assume a dependence in the frequency-rank plot of the form

9 $$\begin{aligned} g(z(r)) = -a \ln (r) + b \end{aligned}$$

with a strictly increasing function *g* and a constant $a>0$. Then, we get

$$\begin{aligned} r = \exp \Big ( -\frac{g(z(r))-b}{a} \Big )\,. \end{aligned}$$

If *g* is differentiable, we have

$$\begin{aligned} \frac{\partial z(r)}{\partial r} = \frac{\partial g^{-1} (-a \ln (r) + b) }{\partial r} = -\frac{1}{g'(g^{-1}(-a\ln (r)+b))} \frac{a}{r}\,. \end{aligned}$$

Since $\delta r \approx p(z(r)) |\delta z|$, we get

$$\begin{aligned} p(z(r)) \propto -\frac{1}{\frac{\partial z}{\partial r}}= g'(g^{-1}(-a\ln (r)+b))\frac{r}{a}= \frac{1}{a}g'(z(r))\exp \Big ( -\frac{g(z(r))-b}{a} \Big ). \end{aligned}$$

Setting $y = z(r)$ in the above relation and taking the logarithm, we find

10 $$\begin{aligned}  \ln (p(y))&\approx \ln (g'(y)) - \frac{g(y)}{a} + C_1 \\&\approx - \ln \Big (\frac{a\nu }{g'(y)}\Big ) - \nu \int _{y_0}^{y} \frac{1}{a\nu } g'(s) \,ds + C_2\,.  \end{aligned}$$

If we compare this last equation with (8), we arrives to the relation (3) between the function *F* of the model and the function *g* describing the frequency rank plot.

### Behavior of $D_t$

Since (4), the behavior of $D_t$ can be obtained starting from the frequency-rank function $r\mapsto z(r)$. For instance, in the case of a pure (generalized) Zipf’s law with $\alpha \ne 1$, we have

$$\begin{aligned} t\approx \int _1^{D_t} z_{max}r^{-\alpha }dr=z_{max}\frac{(D_t^{1-\alpha }-1)}{1-\alpha } \end{aligned}$$

and so, taking into account that $z(D_t)\propto 1$, i.e. $z_{max}\propto D_t^{\alpha }$, we get $D_t\propto t^{1/\alpha }$ for $\alpha <1$ and $D_t\propto t$ for $\alpha >1$. When $\alpha =1$, we find $t\approx D_t \ln (D_t)$ and so $D_t\propto t/\ln (t)$.

When *z*(*r*) exhibits two different behaviors, one for small ranks, say for $r< \xi _t$, and the other for large ranks, say for $r> \xi _t$, the above relation becomes

11 $$\begin{aligned} t\approx \int _1^{\xi _t}z(r)dr +\int _{\xi _t}^{D_t}z(r)dr\,. \end{aligned}$$

In the following, we will study different cases: the first one is observed in the real data sets that we discuss in the present work and the other two have been observed in other papers^43,46–48^.

#### $g(z)=\ln z$ for $z<z_*$ and $g=\sqrt{z}$ for $z>z_*$

Suppose that the frequency-rank plot identifies the following dependence structure:

12 $$\begin{aligned} {\left\{ \begin{array}{ll} \sqrt{z(r)} - \sqrt{z_*} = -a_1 \big [ \ln \big ( \frac{r}{D_t} \big ) -R_* \big ] &{}\text{ for } \ln \big ( \frac{r}{D_t} \big ) < R_* \\ \ln {z(r)} - \ln {z_*} = -a_2 \big [ \ln \big ( \frac{r}{D_t} \big ) -R_* \big ] &{}\text{ for } \ln \big ( \frac{r}{D_t} \big ) > R_*\,, \end{array}\right. } \end{aligned}$$

where $a_1,\,a_2,\, R_*$ and $z_*$ are constants such that $a_i>0$, $R_*<0$ and $z_*=z(\xi _t)$ with $\xi _t=D_te^{R_*}$. This corresponds to

$$\begin{aligned} g(z) = {\left\{ \begin{array}{ll} \ln (z) &{}\text{ for } z < z_*\\ \sqrt{z} &{}\text{ for } z > z_* \end{array}\right. } \end{aligned}$$

and, leveraging (3), we detect the function *F* in the model as

13 $$\begin{aligned} F(x)={\left\{ \begin{array}{ll} {\widehat{\rho }}-\rho + \rho x \quad &{}\text{ for } 1\le x<z_*\\ {\widehat{\rho }}-\rho + \rho \sqrt{z_*x} \quad &{}\text{ for } x\ge z_*\,, \end{array}\right. } \end{aligned}$$

where ${\widehat{\rho }}>0$, $\sqrt{z_*}\rho = 2\nu a_1$, $\rho =\nu a_2>0$ and so

14 $$\begin{aligned} z_*=4\left( \frac{a_1}{a_2}\right) ^2. \end{aligned}$$

From (9) and (12), we get

$$ \begin{aligned} z(r)&={\left\{ \begin{array}{ll} g^{-1}(-a_1 \ln r + b_1) &{}\text{ for } \ln \big ( \frac{r}{D_t} \big )< R_*\\ g^{-1}(-a_2 \ln r + b_2) &{}\text{ for } \ln \big ( \frac{r}{D_t} \big )> R_* \end{array}\right. } \\&= {\left\{ \begin{array}{ll} (-a_1 \ln r + b_1)^2 &{}\text{ for } r < \xi _t = D_t e^{R_*} \\ e^{b_2} r^{-a_2} &{}\text{ for } r > \xi _t = D_t e^{R_*} \,, \end{array}\right. }  \end{aligned}$$

where

15 $$\begin{aligned} -a_1 \ln \xi _t + b_1 = \sqrt{z_*}\qquad \text{ and }\qquad -a_2 \ln \xi _t + b_2 = \ln (z_*). \end{aligned}$$

Now, we recall that

$$\begin{aligned} \int _1^x (-a_1\ln (r)+b_1)^2dr= x\left[ (-a_1\ln (x)+b_1)^2 + 2a_1(-a_1\ln (x)+a_1+b_1)\right] -b_1^2-2a_1^2-2a_1 b_1\,. \end{aligned}$$

Taking in the above integral $x=\xi _t$ and using the first equality in (15), we obtain

16 $$\begin{aligned} \int _1^{\xi _t}z(r)dr= \xi _t (z_*+2a_1^2+2a_1\sqrt{z_*}) + O(\ln ^2(\xi _t)) = D_t e^{R_*} z_*[1+\rho ^2/(2\nu ^2)+\rho /\nu ] + O(\ln ^2(D_t)) \,. \end{aligned}$$

For the second integral, assuming $a_2=\rho /\nu \ne 1$ and using the second equality in (15), we get

17 $$ \begin{aligned} \int _{\xi _t}^{D_t}z(r)dr&= \frac{e^{b_2}}{1-a_2} [ D_t^{1-a_2} - \xi _t^{1-a_2} ] = D_t \frac{z_* }{1-a_2}( e^{a_2R_*} - e^{R_*}) \\&= D_t \frac{z_* e^{R_*}}{(\rho /\nu -1)} ( 1 - e^{R_*(\rho /\nu -1)}) \,,  \end{aligned}$$

while for $a_2 = \rho /\nu =1$, we get

18 $$\begin{aligned} \int _{\xi _t}^{D_t}z(r)dr= e^{b_2} (\ln (D_t) - \ln (\xi _t) )= -z_* R_* D_t e^{R_*}\,. \end{aligned}$$

From (11), (16), (17) and (18), we can conclude that $D_t\propto t$. This means that we have the Heaps’ law with exponent $\gamma =1$.

#### Zipf’s law with two different coefficients

Suppose that the frequency-rank plot identifies a “double” Zipf’s law, that is the following dependence structure:

19 $$\begin{aligned} {\left\{ \begin{array}{ll} \ln {z(r)} - \ln {z_*} = -a_1 \big [ \ln \big ( \frac{r}{D_t} \big ) -R_* \big ] &{}\text{ for } \ln \big ( \frac{r}{D_t} \big ) < R_* \\ \ln {z(r)} - \ln {z_*} = -a_2 \big [ \ln \big ( \frac{r}{D_t} \big ) -R_* \big ] &{}\text{ for } \ln \big ( \frac{r}{D_t} \big ) > R_*\,, \end{array}\right. } \end{aligned}$$

where $a_1,\,a_2$ and $R_*$ are constants such that $a_i>0$, $a_1\ne a_2$ (typically $a_1<a_2$), $R_*<0$ and $z_*=z(\xi _t)$ with $\xi _t=D_te^{R_*}$. This kind of dependence was observed in^43,46,48^ and, according to our model (see (3)), it corresponds to

$$\begin{aligned} F(x)={\left\{ \begin{array}{ll} {\widehat{\rho }}-\rho _1+\rho _1 x \quad &{}\text{ for } 1\le x<z_*\\ {\widehat{\rho }}-\rho _1+\rho _2 x + (\rho _1-\rho _2)z_* \quad &{}\text{ for } x\ge z_*\,, \end{array}\right. } \end{aligned}$$

where $\rho _1=\nu a_2\ne \rho _2 = \nu a_1$ (typically $\rho _2<\rho _1$) and ${\widehat{\rho }}>0$.

From (9) and (19), we get

$$ \begin{aligned} z(r)&={\left\{ \begin{array}{ll} g^{-1}(-a_1 \ln r + b_1) &{}\text{ for } \ln \big ( \frac{r}{D_t} \big )< R_*\\ g^{-1}(-a_2 \ln r + b_2) &{}\text{ for } \ln \big ( \frac{r}{D_t} \big )> R_* \end{array}\right. } \\&= {\left\{ \begin{array}{ll} e^{b_1} r^{-a_1} &{}\text{ for } r < \xi _t = D_t e^{R_*} \\ e^{b_2} r^{-a_2} &{}\text{ for } r > \xi _t = D_t e^{R_*} \,, \end{array}\right. }  \end{aligned}$$

where $b_1 = \ln {z_*} + a_1 \ln \xi _t$ and $b_2 = \ln {z_*} + a_2 \ln \xi _t$. Therefore, we have

20 $$\begin{aligned} \int _1^{\xi _t}z(r)dr= {\left\{ \begin{array}{ll} \frac{z_*}{1-a_1}(D_t e^{R_*}- D_t^{a_1} e^{R_*a_1}) \qquad &{}\text{ for } a_1=\rho _2/\nu \ne 1\\ z_*e^{R_*}D_t(\ln (D_t)+R_*)\qquad &{}\text{ for } a_1=\rho _2/\nu =1\,. \end{array}\right. } \end{aligned}$$

For the second integral, as before, we have

21 $$\begin{aligned} \int _{\xi _t}^{D_t}z(r)dr= {\left\{ \begin{array}{ll} D_t\frac{z_* e^{R_*}}{a_2-1}(1-e^{R_*(a_2-1)}) \qquad &{}\text{ for } a_2=\rho _1/\nu \ne 1\\ -z_*R_*D_t e^{R_*}\qquad &{}\text{ for } a_2=\rho _1/\nu = 1\,. \end{array}\right. } \end{aligned}$$

From (11), (20), (21), we can conclude that the asymptotic behavior of $D_t$ is ruled by the value of $a_1=\rho _2/\nu$: $D_t\propto t$ when $a_1<1$, $D_t\propto t/\ln (t)$ when $a_1=1$ and $D_t\propto t^{1/a_1}$ when $a_1>1$.

#### Frequency-rank plot with exponential decay

Suppose that the frequency-rank plot identifies the following dependence structure:

22 $$\begin{aligned} \ln (z(r)) = -a r + b,\quad \text{ that } \text{ is }\quad z(r)=C e^{-ar}\,, \end{aligned}$$

where $a>0$ and $C=e^b\propto e^{aD_t}$. This is the case observed in^47^. The corresponding behavior of $D_t$ is given by the relation (11), that is

$$\begin{aligned} t\approx -\frac{C}{a}(e^{-a D_t}-e^{-a})=\frac{1}{a}(e^{a(D_t-1)}-1)\approx \frac{1}{a}e^{aD_t}\,, \end{aligned}$$

that implies $D_t\propto \ln (t)$. These behaviors for *z*(*r*) and $D_t$ can be achieved by the introduced model with $F(x)=\rho (x+1)\ln (x+1)$ and $\rho >0$. Indeed, starting from (22) and employing an adaption of the argument used in Section 3.2, we find $p(z(r))\propto \frac{1}{a z(r)}$ and so $\ln (p(y))\approx -\ln (y)$. On the other hand, when inserting the above function *F* in (8), we find $\ln (p(k))\approx -\ln (k+1)-(1+\nu /\rho )\ln (\ln (k+1))+C''\propto -\ln (k)$. The relation (3) is satisfied with $g(z)=\ln (\ln (z))$ and $\rho =a\nu$.

### Empirical analyses: parameters estimation

**Figure 7:**
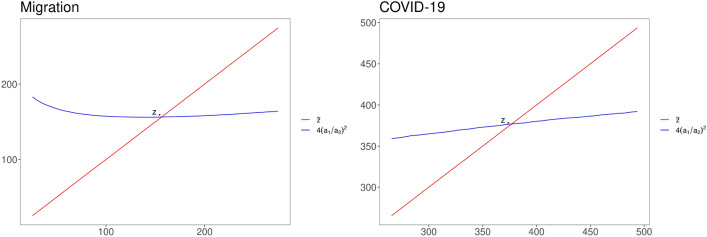
Estimation of the parameter $z_*$ based on Eq. (14): (Left) Migration; (Right) COVID-19.

For each ${\tilde{z}}$, we fit

$$\begin{aligned} {\left\{ \begin{array}{ll} \sqrt{z(r)}= -a_1 \big [ \ln \big ( \frac{r}{D_T} \big ) + b_1 \big ] &{}\text{ for } z(r)> {\tilde{z}} \\ \ln {z(r)} = -a_2 \big [ \ln \big ( \frac{r}{D_T} \big ) + b_2 \big ] &{}\text{ for } z(r)< {\tilde{z}} \end{array}\right. } \end{aligned}$$

and we compute the corresponding quantity $4(a_1/a_2)^2$ (see (14)). Finally, as shown in Fig. 7, we choose $z_*={\tilde{z}}$ such that ${\tilde{z}}=4(a_1/a_2)^2$ and we set $\rho /\nu$ equal to the corresponding $a_2$.

## References

[1] Armano G, Javarone MA (2017). The beneficial role of mobility for the emergence of innovation. Sci. Rep..

[2] Arthur, W. B. The nature of technology: what it is and how it evolves (Free Press, 2009).

[3] Fink TMA, Reeves M, Palma R, Farr RS (2017). Serendipity and strategy in rapid innovation. Nat. Commun..

[4] Gooday, G., & Ziman, J. Technological innovation as an evolutionary process. *Br. J. History Sci.* **34** (2001).

[5] O’Brien, M. & Shennan, S. Innovation in cultural systems contributions from evolutionary anthropology. *Vienna Series in Theoretical Biology* (2010).

[6] Puglisi A, Baronchelli A, Loreto V (2008). Cultural route to the emergence of linguistic categories. Proc. Natl. Acad. Sci..

[7] Reader, S. & Laland, K. *An introduction*. (Animal innovation, 2003).

[8] Rogers, E., Singhal, A. & Quinlan, M. *Diffusion of innovations* (Taylor and Francis, 2019). https://www.scopus.com/inward/record.uri?eid=2-s2.0-85070280457&doi=10.4324%2f9780203710753-35&partnerID=40&md5=ef28257e8b71b92678b78730d4d089f9 Cited By 18.

[9] Rzhetsky A, Foster JG, Foster IT, Evans JA (2015). Choosing experiments to accelerate collective discovery. Proc. Natl. Acad. Sci..

[10] Saracco F, Di Clemente R, Gabrielli A, Pietronero L (2015). From innovation to diversification: A simple competitive model. PLoS ONE.

[11] Sole R (2013). The evolutionary ecology of technological innovations. Complexity.

[12] Thurner S, Klimek P, Hanel R (2010). Schumpeterian economic dynamics as a quantifiable model of evolution. N. J. Phys..

[13] Hansen B, Pitman J (2000). Prediction rules for exchangeable sequences related to species sampling. Stat. Probab. Lett..

[14] Pitman J (1996). Some developments of the bBackwell–Macqueen urn scheme. Lecture Notes-Monogr. Ser..

[15] Zabell S (1992). Predicting the unpredictable. Synthese.

[16] Pitman J (1995). Exchangeable and partially exchangeable random partitions. Probab. Theory Relat. Fields.

[17] Pitman, J. *Combinatorial Stochastic Processes*. Ecole d’Eté de Probabilités de Saint-Flour XXXII (Springer, 2006).

[18] Blackwell D, MacQueen JB (1973). Ferguson distributions via Pólya urn schemes. Ann. Stat..

[19] Ferguson TS (1973). A Bayesian analysis of some nonparametric problems. Ann. Stat..

[20] Hoppe FM (1987). The sampling theory of neutral alleles and an urn model in population genetics. J. Math. Biol..

[21] James, L. F. *Large sample asymptotics for the two-parameter Poisson-Dirichlet process. Pushing the Limits of Contemporary Statistics: Contributions in Honor of Jayanta K. Ghosh* (Institute of Mathematical Statistics, Beachwood, Ohio, USA, 2008).

[22] Pitman J, Yor M (1997). The two-parameter Poisson–Dirichlet distribution derived from a stable subordinator. Ann. Appl. Probab..

[23] Teh, Y. W. A hierarchical bayesian language model based on pitman-yor processes. *Proceedings of COLING/ACL 2006* (2006). https://ci.nii.ac.jp/naid/10019458975/en/.

[24] Bassetti F, Crimaldi I, Leisen F (2010). Conditionally identically distributed species sampling sequences. Adv. Appl. Probab..

[25] Berti P, Pratelli L, Rigo P (2004). Limit theorems for a class of identically distributed random variables. Ann. Probab..

[26] Heaps, H. S. *Information Retrieval-Computational and Theoretical Aspects* (Academic Press, 1978).

[27] Herdan, G. *Type-token Mathematics: A Textbook of Mathematical Linguistics*. Janua linguarum. series maior. no. 4 (Mouton en Company, 1960). https://books.google.it/books?id=jJhkQwAACAAJ.

[28] Zipf GK (1929). Relative frequency as a determinant of phonetic change. Harv. Stud. Class. Philos..

[29] Zipf GK (1935). The Psychobiology of Language.

[30] Zipf GK (1949). Human Behavior and the Principle of Least Effort.

[31] Tria, F., Crimaldi, I., Aletti, G. & Servedio, V. Taylor’s law in innovation processes. *Entropy* **22**, 573 (2020). 10.3390/e22050573 PMC7517092 33286342

[32] Tria, F., Loreto, V. & Servedio, V. D. P. Zipf’s, Heaps’ and Taylor’s Laws are Determined by the Expansion into the Adjacent Possible. *Entropy* **20**, (2018). 10.3390/e20100752 PMC7512314 33265841

[33] Tria, F., Loreto, V., Servedio, V. D. P. & Strogatz, S. H. The dynamics of correlated novelties. *Sci. Rep.* **4**, (2014). 10.1038/srep05890 PMC5376195 25080941

[34] Kauffman SA (2000). Investigations.

[35] Iacopini I, Milojević SCV, Latora V (2018). Network dynamics of innovation processes. Phys. Rev. Lett..

[36] Lü, L., Zhang, Z.-K. & Zhou, T. Zipf’s law leads to heaps’ law: Analyzing their relation in finite-size systems. *PLOS ONE* **5**, 1–11 (2010). 10.1371/journal.pone.0014139. 10.1371/journal.pone.0014139 PMC2996287 21152034

[37] Corominas-Murtra B, Hanel R, Thurner S (2015). Understanding scaling through history-dependent processes with collapsing sample space. Proc. Natl. Acad. Sci..

[38] Cubero RJ, Jo J, Marsili M, Roudi Y, Song J (2019). Statistical criticality arises in most informative representations. J. Stat. Mech..

[39] Mitzenmacher M (2003). A brief history of generative models for power law and lognormal distributions. Internet Math..

[40] Newman, M. E. J. Power laws, Pareto distributions and Zipf’s law. *Contemp. Phys.* **46**, 323–351 (2005).

[41] Simon H (1955). On a class of skew distribution functions. Biometrika.

[42] Zanette, D. & Montemurro, M. Dynamics of text generation with realistic zipf’s distribution. *J. Quant. Linguist.* **12**, 29 (2005).

[43] Cancho, R. F. & Solé, R. V. Two regimes in the frequency of words and the origins of complex lexicons: Zipf’s law revisited. *J. Quant. Linguist.* **8**, 165–173 (2001).

[44] Cattuto C, Loreto V, Pietronero L (2007). Semiotic dynamics and collaborative tagging. Proc. Natl. Acad. Sci..

[45] Clauset A, Shalizi CR, Newman MEJ (2009). Power-law distributions in empirical data. SIAM Rev..

[46] Gerlach M, Altmann EG (2013). Stochastic model for the vocabulary growth in natural languages. Phys. Rev. X.

[47] Lü, L., Zhang, Z.-K. & Zhou, T. Deviation of zipf’s and heaps’ laws in human languages with limited dictionary sizes. *Sci. Rep.* **3**, 1082 (2013). 10.1038/srep01082 PMC3558701 23378896

[48] Petersen A, Tenenbaum J, Havlin S, Stanley H, Perc M (2012). Languages cool as they expand: Allometric scaling and the decreasing need for new words. Sci. Rep..

[49] Guille A, Hacid H, Favre C, Zighed D (2013). Information diffusion in online social networks: A survey. ACM SIGMOD Rec..

[50] Kobayashi, R. & Lambiotte, R. Tideh: Time-dependent hawkes process for predicting retweet dynamics. In *Proceedings of the 10th International Conference on Web and Social Media, ICWSM 2016*, 191–200 (AAAI Press, 2016). https://www.aaai.org/ocs/index.php/ICWSM/ICWSM16/paper/view/13026. Cited By 55; Conference of 10th International Conference on Web and Social Media, ICWSM 2016 ; Conference Date: 17 May 2016 Through 20 May 2016; Conference Code:122446.

[51] ten Thij, M. & Bhulai, S. Modelling trend progression through an extension of the polya urn process. In Wierzbicki, A., Brandes, U., Schweitzer, F. & Pedreschi, D. (eds.) *Advances in Network Science*, vol. 9564, 57–67 (Springer Verlag, 2016). 12th International Conference and School of Network Science, NetSci-X 2016 ; Conference date: 11-01-2016 Through 13-01-2016.

[52] ten Thij, M. *et al.* Modelling of trends in twitter using retweet graph dynamics. *Lecture Notes in Computer Science* **2014**, 132–147 (2014). Proceedings title: 11th International Workshop, WAW 2014, Beijing, China, December 17-18, 2014, Proceedings Publisher: Springer International Publishing ISBN: 978-3-319-13122-1 Editors: F.C. Graham, P. Pralat, A. Bonato; 11th Workshop on Algorithms and Models for the Webgraph ; Conference date: 17-12-2014 Through 18-12-2014.

[53] Caldarelli G, De Nicola R, Del Vigna F, Petrocchi M, Saracco F (2020). The role of bot squads in the political propaganda on Twitter. Commun. Phys..

[54] Caldarelli, G., de Nicola, R., Petrocchi, M., Pratelli, M. & Saracco, F. Analysis of online misinformation during the peak of the covid-19 pandemics in italy (2020). 10.1140/epjds/s13688-021-00289-4 PMC8258478 34249599

